# Surgery/anesthesia may cause monocytes to promote tumor development

**DOI:** 10.1186/s10020-025-01213-6

**Published:** 2025-05-07

**Authors:** Yang-Yang Wang, Rui-Lou Zhu, En-Qiang Chang, Xiao-Zhuan Liu, Guang-Zhi Wang, Ning-Tao Li, Wei Zhang, Jun Zhou, Ming-Yang Sun, Xin Zou, Jie Hao, Jia-Qiang Zhang

**Affiliations:** 1 Department of Anesthesiology and Perioperative Medicine, Center for Clinical Single Cell Biomedicine, Henan Provincial People’s Hospital, People’s Hospital of Zhengzhou University, Zhengzhou, 450003 Henan China; 2 Center for Clinical Single Cell Biomedicine, Henan Provincial People’s Hospital, People’s Hospital of Zhengzhou University, Zhengzhou, 450003 Henan China; 3 Digital Diagnosis and Treatment Innovation Center for Cancer, Institute of Translational Medicine, Shanghai Jiao Tong University, Shanghai, 200240 China; 4 Shanghai Key Laboratory of Plant Functional Genomics and Resources, Shanghai Chenshan Botanical Garden, Shanghai, 201602 China

**Keywords:** General anesthesia, PBMCs, Monocytes, ScRNA-seq, Tumor development

## Abstract

**Background:**

The immune system of patients undergoing major surgery usually has obvious immune responses during the perioperative period, and the patient’s immune status would affect the patient’s prognosis. In this study single-cell sequencing technology was used to investigate the effect of surgery/anesthesia on peripheral blood mononuclear cells (PBMCs) in depth during the perioperative period.

**Methods:**

We performed an in-depth analysis of our previously published data, which included a total of 4 patients were recruited in this study. Their peripheral blood samples were collected pre operation, 0, 24, and 48 h post operation, and then PBMCs were extracted, followed by single cell sequencing. The results of sequencing were analyzed with R packages seurat and scSTAR. Finally, RT-PCR technology was used to verify the expression of key genes in monocyte.

**Results:**

The ratio of CD4^+^ and CD8^+^ T cells and Tregs showed little change, and the function of CD4^+^ and CD8^+^ T cells recovered soon. The function of Treg had not been restored 48 h post operation. Non-classical monocyte was impressed after surgery and showed no recovery trend within 48 h. Similar to scRNA-seq, the expression levels of MDM2 and SESN1 in patients with tumor increased significantly after surgery.

**Conclusions:**

Surgery/anesthesia had little effect on CD4^+^ and CD8^+^ T cells, and continued to affect the functional changes of Treg. It had more impact on monocytes, which may cause them to promote tumor development to a certain extent.

**Supplementary Information:**

The online version contains supplementary material available at 10.1186/s10020-025-01213-6.

## Introduction

Although many physicians have been trying various non-surgical treatments for tumors, surgical resection remains the first-line treatment for solid tumors. The effect of general anesthesia during surgery on the prognosis of cancer patients remains controversial (Buggy et al. 2015). And it is necessary to conduct in-depth clinical research to determine the impact of anesthesia on immunity and its role in cancer treatment.

Malignant tumors account for a large proportion of patients undergoing surgery, and the postoperative immune status of these patients was related to their prognosis. There have been many studies on the effect of anesthesia on the immune system, and more and more pre-clinical laboratory data showed that general anesthetics had the ability to affect the hallmarks of cancers related to tumorigenesis and metastasis (Perry et al. 2019). The role of opioids in the development of cancer metastasis and recurrence was unclear, and seemed to vary depending on the type of cancer cell in question (Juneja 2014). However, previous studies were highly restricted, which were mostly limited to a certain kind of anesthetics or certain cell ratios or changes in cytokines (Müller et al. 2021).

In our preliminary work, we collected peripheral blood samples from patients at 4 perioperative time points, extracted PBMCs and performed single-cell sequencing. At the same time, an atlas of dynamic peripheral blood mononuclear cell (PBMC) landscapes in human perioperative anesthesia/surgery was illustrated (Wang et al. 2022) with no further data mining was carried out. In this study, we try to illustrate the effect of the process of surgery/anesthesia on various types of immune cells and find the influence of immune changes induced by anesthesia on cancer patients.

Our study may demonstrate the dynamic changes of PBMC clusters during the perioperative period and showed that T cells recovered soon within 48 h and non-classical monocyte was impressed after surgery and showed no recovery trend within 48 h. Our study also provides some clues to improve the prognosis of patients undergoing general anesthesia for tumor resection surgery.

## Materials and methods

### Patient information and analytical strategies

The characteristics of the patients were collected, including basic information, surgery type, and the route of anesthesia, as well as the results of routine blood tests conducted at each time point. To study changes in PBMCs from patients during the perioperative period, peripheral blood samples were collected at four time points, namely before anesthetic administration (pre operation), immediately after operation (0 h post operation), 24 h post operation, and 48 h post operation. The data of all four patients were combined, and changes in PBMCs at each time point were analyzed. None of the patients received immune-related treatment from the preoperative period to 48 h post operation. The basic information of each patient was shown in the supplementary materials (Table S1).

### Sorting of monocytes with CD14 microbeads

First, peripheral blood mononuclear cell (PBMC) number should be calculated and centrifuge cell suspension at 300 × g for 10 min. Supernatant was aspirated completely. Then, resuspend cell pellet in 80 µL of buffer per 10⁷ total cells. Add 20 µL of CD14 Microbeads per 10⁷ total cells and mix well and incubate for 15 min at 4 °C. Next, wash cells by adding 1−2 mL of buffer per 10⁷ cells and centrifuge at 300 × g for 10 min. Supernatant was aspirated completely and resuspend up to 10⁸ cells in 500 µL of buffer. Subsequently, magnetic sorting was performed by LS columns. Put column in the magnetic field of a suitable MACS separator. Prepare column by rinsing with the appropriate amount of 3 ml buffer (BSA Stock Solution: autoMACS™ Rinsing Solution = 1:20). After that, remove column from the separator and place it on a suitable collection tube. Pipette the 5 ml buffer onto the column and immediately flush out the magnetically labeled cells by firmly pushing the plunger into the column.

### Real-time PCR

Total RNA was isolated from monocytes by using Trizol reagent (Takara), and the cDNA was synthesized with the PrimeScript^™^ RT Master Mix (Takara) according to the manufacturer’s protocol. Quantitative PCR was performed with the SYBRTM Select Master Mix (Applied Biosystems) using the Applied Biosystems Real-Time PCR system. The relative mRNA level values were normalized to β-actin to calculate fold-changes in expression. All primers were list in Table 1.

**Table 1:** Primers’ list

Gene symbol	Forward primer (5′−3′)	Reverse primer (5′−3′)
CDKN1 A	CGATGGAACTTCGACTTTGTCA	GCACAAGGGTACAAGACAGTG
SESN1	TCACACACTATCATTCTCTTGCC	ACATTCCTGTAACTGCCTCATCT
MDM2	GGCAGGGGAGAGTGATACAGA	GAAGCCAATTCTCACGAAGGG
GADD45B	TACGAGTCGGCCAAGTTGATG	GGATGAGCGTGAAGTGGATTT

### Statistical analysis and data visualization

All statistical analyses were performed in R v4.1.0. The comparison of gene expression between two different groups was performed using Wilcoxon rank-sum tests. Gene expression associated with cancers were analyzed with the Kaplan–Meier methods (Nagy et al. 2021). The scRNA-seq data was interpreted using Seurat (v 4.0.3) and scSTAR (v 1.0) (Jie et al. 2022). For all 16 sample, we used Seurat R package v4.0.3 to screen low quality cells. Specifically, the cells with 200 < nFeature_RNA < 4000, nCount_RNA < 20,000 and percent.Mito < 0.1 were preserved. The violin plots for quality control are shown in Fig QC in supplementary materials. We used Seurat CCA method with anchor. Features = 2000 to integrate all samples to remove batch effects. The differential genes were identified by using Seurat FindAllMarkers function with log FC > 0.25. The cells were clustered using Seurat FindClusters with resolution = 0.2. The resolution was set to 0.2 for the original data analysis to distinguish different cell subtypes. For the scSTAR processed data, the resolutions were set to 0.1 (CD8 T), 0.15 (CD4 T), 0.3 (Treg), 0.3 (monocyte) to discriminate the cells from different timepoints. When plotting the heatmap of differentially expressed genes, we performed normalization by calculating the Z-score. When the value of *p* was less than 0.05, it was taken to indicate statistical significance.

## Results

### Alteration of PBMCs from the pre-operation to 48 h after surgery

**Fig. 1:**
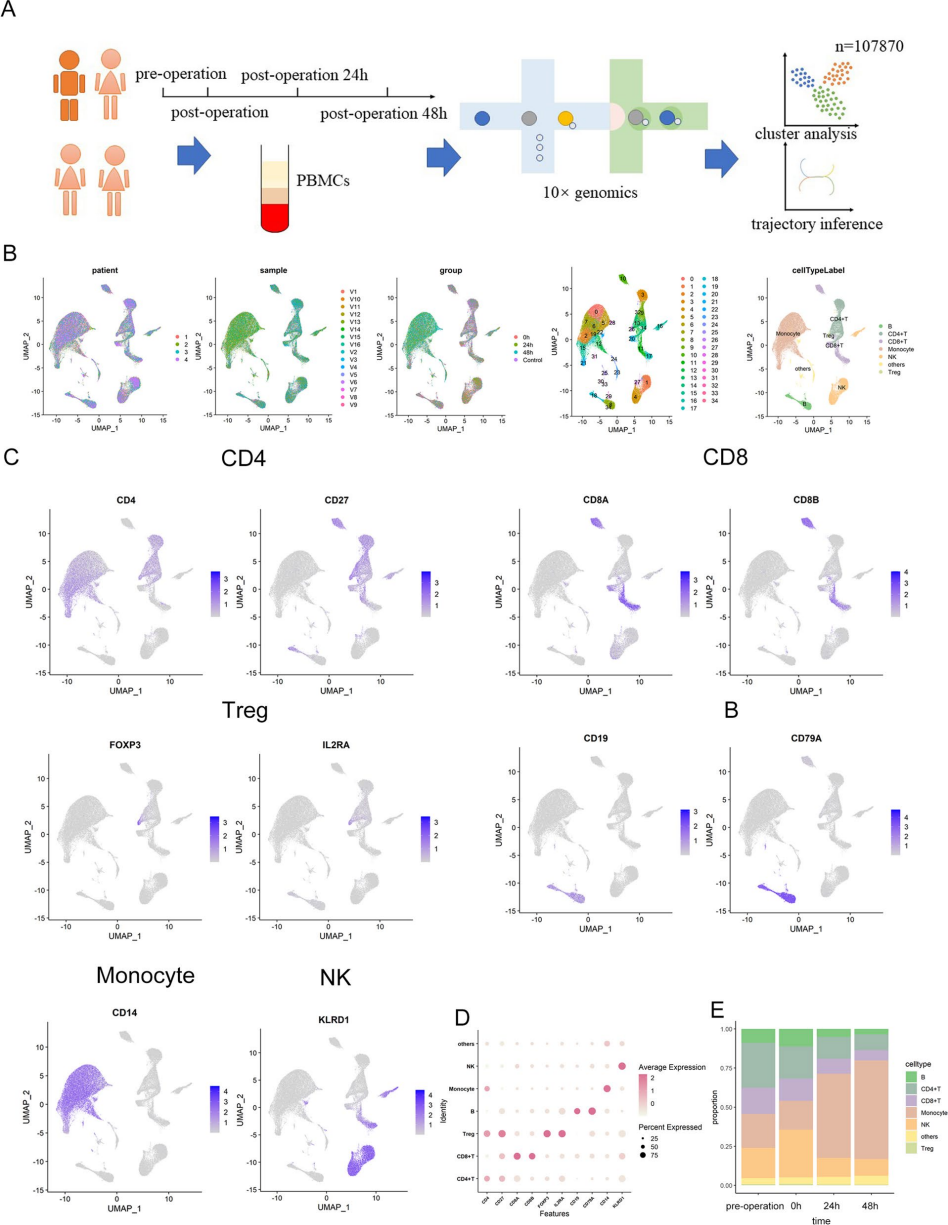
Clustering of PBMCs during the perioperative period. **A** Scheme of overall study design. **B**. UMAP plots of 107,870 single PBMCs from 4 patients at 4 time points, showing the formation of 34 clusters which was annotated as B cells, CD4 ^+^ T cells, CD8 ^+^ T cells, monocytes, NK cells, Treg cells and others. V1-V16 represent 16 samples from 4 patients at 4 time points. **C**. Expression level of CD4, CD27, CD8 A, CD8B, CD14, CD19, CD79 A, FOXP3, IL2RA and KLRD1 across all PBMCs illustrated in UMAP plots. **D**. The bubble chart represented the marker expression of various cell types. **E**. Proportion of B cells, CD4 ^+^ T cells, CD8 ^+^ T cells, monocytes, NK cells, Treg cells and others at 4 time points

To acquire the landscape of PBMCs perioperative variation, blood collection was performed with 4 patients accepted general anesthesia at 4 time points, and 107,870 single PBMCs from 16 samples were sequenced with 10 × Genomics (Fig. 1A). PBMCs from all samples were divided into 35 clusters which could be annotated as 7 types of immune cells: B cells, CD4^+^ T cells, CD8^+^ T cells, monocytes, NK cells, Treg cells and others (Fig. 1B). The differentially expressed genes for each cluster are listed in Table S3, which can be found in the supplementary materials. According to the results illustrated in the UMAP, cells of each cluster from 4 patients and 4 time points were mixed (Fig. 1B). CD4^+^ T cells were labeled according the expression of CD4 and CD27. CD8^+^ T cells were labeled according to the expression of CD8 A and CD8B. Monocytes were labeled based on the expression of CD14. B cells were labeled according to the expression of CD19 and CD79 A. Treg cells were annotated based on the expression of FOXP3 and IL2RA. NK cells were labeled according to the expression of KLRD1 (Fig. 1C). Proportions B cells and NK cells increased promptly (0 h after surgery) and decreased 24 and 48 h post operation. Proportions of CD4^+^ T cells and CD8^+^ T cells deceased after surgery and until 48 h post operation they still showed no increasing trend. Monocytes showed a late and profound increase after 24 h post operation (Fig. 1D).

### Anesthesia has limited effect on CD8^+^ T cells

**Fig. 2:**
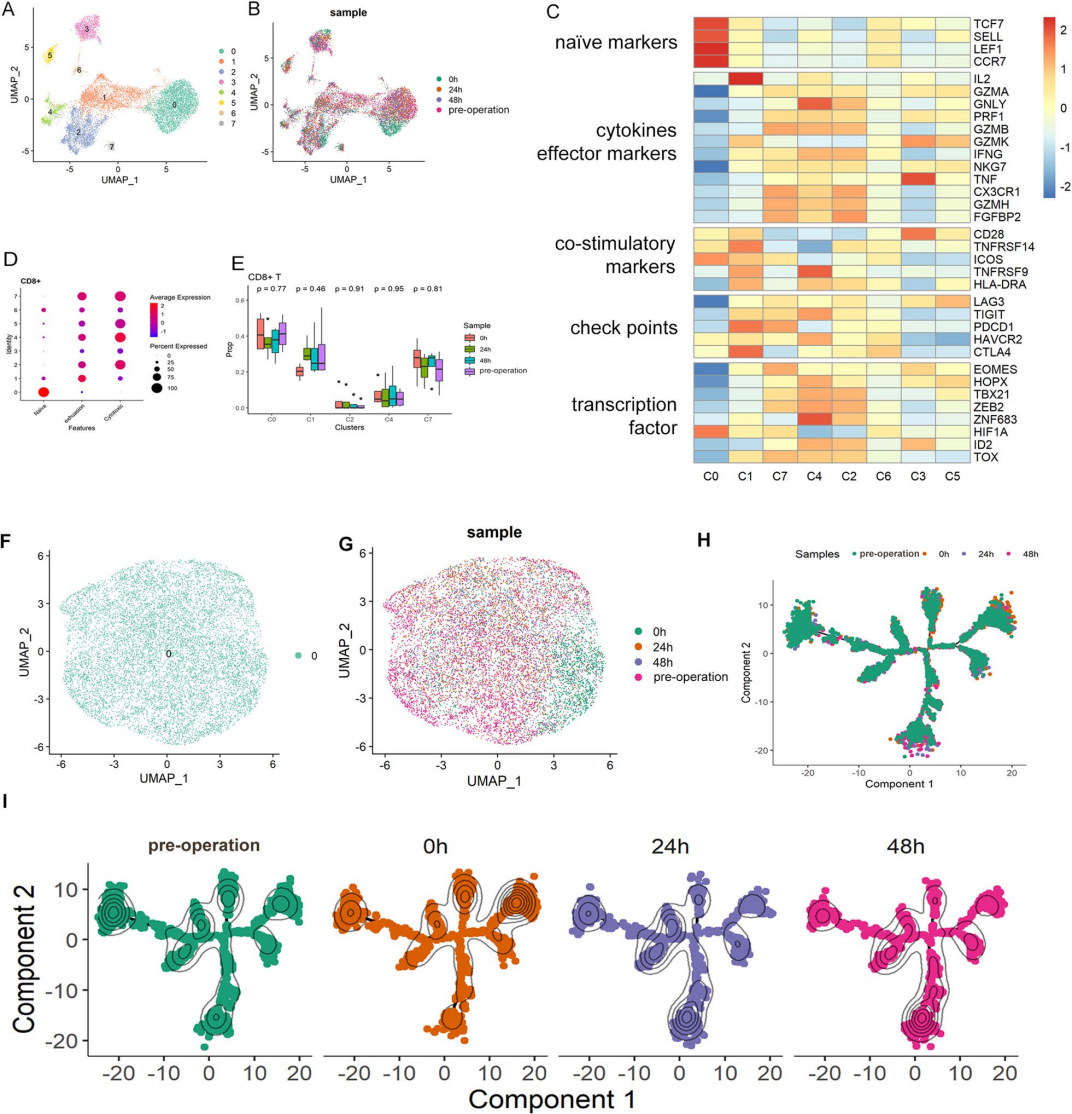
Clustering of CD8 ^+^ T cells during the perioperative period and scSTAR reveals a stimulus pattern of anesthesia-related CD8 ^+^ T cells dynamics. **A** UMAP plots of CD8 ^+^ T cells which were classified as 8 clusters. **B** UMAP plots of CD8 ^+^ T cells from 4 time points. **C** Heatmap of Z-score normalized log2(count + 1) expression of selected T cell function-associated genes in each cell cluster. **D** Dot plot of representative naïve, exhaustive, and cytotoxic signatures, Z-score normalized log2(count + 1). **E** Changes of proportions of c0, c1, c2, c4, and c7 in CD8 ^+^ T cell during the perioperative period. **F** UMAP plot of 1 cell cluster processed with scSTAR. **G** UMAP plot of samples from 4 time points. **H**, **I** Pseudotime trajectory illustrates the transition and differentiation of monocyte clusters from pre operation to 48 h post operation with scSTAR processed data

On expression data, CD8^+^ T cells can be divided into 8 clusters (Fig. 2A), which did not have time specific patterns (Fig. 2B). According to known gene markers of T cell functions, CD8 ^+^ T cells can be categorized into several cell clusters, including naive, cytotoxic, and exhausted T cells (Fig. 2C, Table S4). C0 exhibited the characteristics of naive, in which the expression levels of TCF7, SELL, LEF1, and CCR7 were high, and the expression levels of marker genes of cytotoxic and effector T cells were very low. C1, C4, and C7 highly expressed checkpoint molecule genes, including LAG3, TIGIT, PDCD1, HAVCR2, and CTLA4, which represented the characteristics of exhaustive T cells. C2 highly expressed cytotoxic and effector cell marker genes, including GNLY, GAMB, IFNG, CX3 CR1, GZMH, and FGFBP2, so C2 was identified as cytotoxic T cells. We used the recognized characteristics of naive, exhaustion and cytotoxic in T cells to calculate a transcriptional score based on the gene expression level of each cell cluster (Guo et al. 2018). Such annotation was further confirmed by evaluating the score levels of naïve, exhaustive and cytotoxic marker panels over cell clusters, respectively (Fig. 2D). The CD8^+^ T subcluster compositions of different time points were illustrated in Fig. 2E, which illustrates there was no cell subcluster composition change during perioperative period. It means that there is no difference in the proportion of the CD8 ^+^ T cell subgroups at different time points.

Besides cell subcluster composition, there is no obvious change in the state of CD8^+^ T cells before and after anesthesia. We used scSTAR algorithm to investigate cell dynamics by comparing pre operation (as control) and post operation (combined 0, 24, 48 h as case) CD8^+^ T cells, and found no cell cluster specific to any time point. The processed data were highly homogeneous (Fig. 2F), and the cells from different time points were clustered together (Fig. 2G). Such conclusion was also confirmed by trajectory analysis, which illustrated the cells from each time points distributed across the whole trajectory (Fig. 2H, I).

### CD4^+^ T cells showed prompt response during the perioperative period

**Fig. 3:**
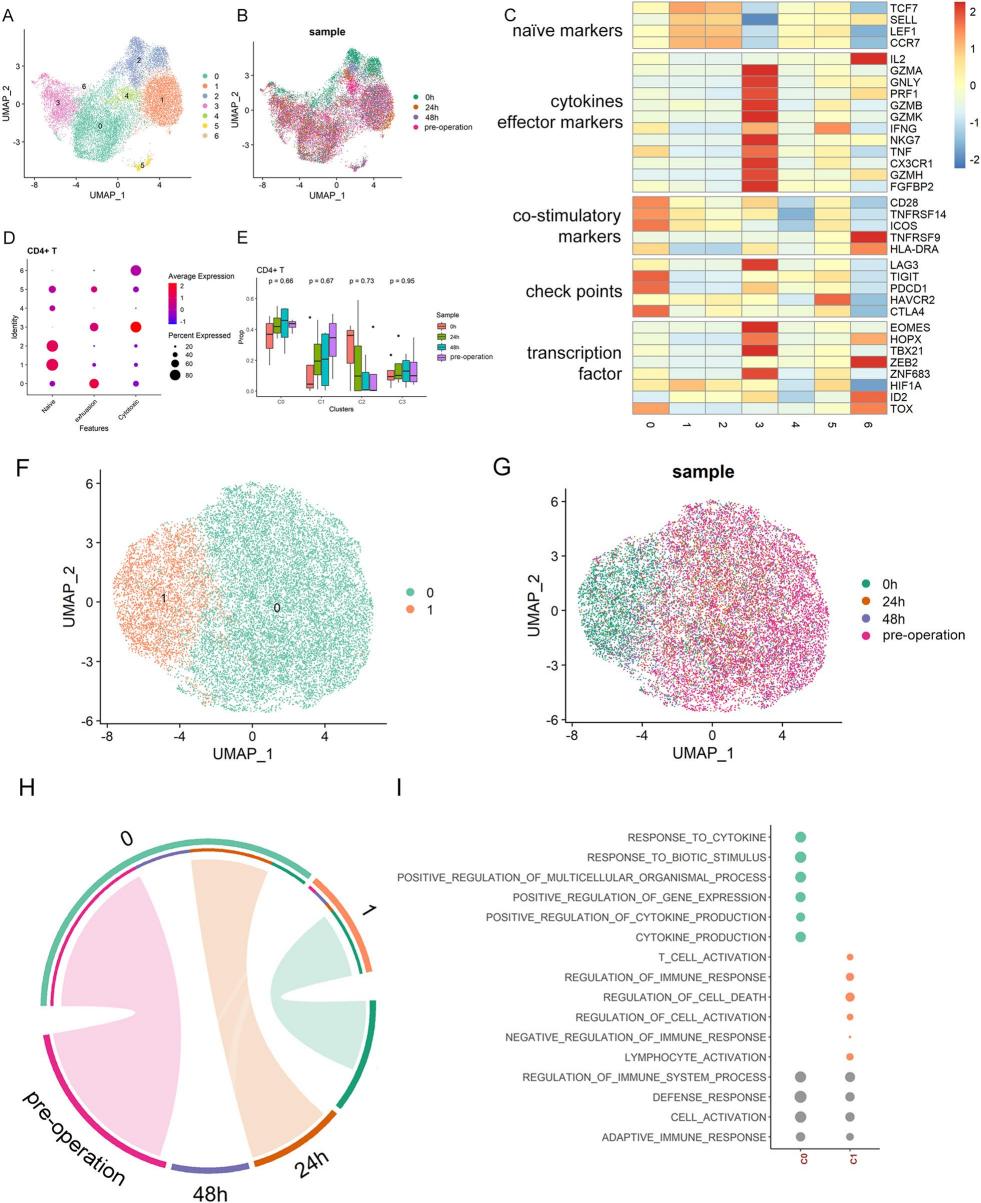
Clustering of C4 + Th cells during the perioperative period and scSTAR reveals a stimulus pattern of anesthesia-related CD4 + Th cells dynamics. **A** UMAP plots of CD4 + Th cells which were classified as 7 clusters. **B** UMAP plots of CD4 + Th cells from 4 time points. **C** Heatmap of Z-score normalized log2(count + 1) expression of selected T cell function-associated genes in each cell cluster. **D** Dot plot of representative naïve, exhaustive, and cytotoxic signatures, Z-score normalized log2(count + 1). **E** Changes of proportions of c0, c1, c2, and c3 in CD4 + Th cells during the perioperative period. **F** UMAP plot of 2 cell clusters of CD4 + Th cells processed with scSTAR. **G** UMAP plots of samples from 4 time points. **H** The association between scSTAR cell clustering results and time points using hypergeometric test, i.e., meaningful cells of C1 were derived from samples at 0 h post operation. **I** GO enrichment of marker genes of c0 and c1 processed with scSTAR

The 16,700 CD4^+^ T helper (Th) cells were divided into 7 clusters based on expression profiles (Fig. 3A, B and Table S5). According to the expression of canonical markers, C1 and C2 were annotated as naïve, C0 as exhausted and C3 as cytotoxic T cells. C0 highly expressed checkpoint molecule genes, including TIGIT, PDCD1, and CTLA4, which represented the characteristics of exhaustive T cells. C1 and C2 exhibited the characteristics of naive, in which the expression levels of TCF7, SELL, LEF1, and CCR7 were high, and the expression levels of marker genes of cytotoxic and effector T cells were very low. C3 highly expressed cytotoxic and effector cell marker genes, including GZMA, GNLY, PRF1, GAMB, MKG7, TNF, CX3 CR1, GZMH, and FGFBP2, so C3 was identified as cytotoxic T cells (Fig. 3C). Consistent to Fig. 3C, the cell subcluster annotation results were further confirmed by calculating the transcriptional scores of naive, exhaustion and cytotoxic in T cells of each cell cluster (Fig. 3D), where the marker list for score calculation were as described in Zheng et al. (2020). However, we did not find significant changes in CD4 Th subcluster compositions during perioperative period (Fig. 3E).

Next, CD4^+^ Th cell dynamics were profiled using scSTAR by comparing the before and after anesthesia. It was observed that the cells can be categorized into 2 clusters (Fig. 3F, G; Table S6), one was dominated by 0 h cells and the other was composed of cells from other time points (Fig. 3H), which indicated that CD4^+^ Th cells responded promptly and recovered soon within 24 h. GO analysis of the regulated genes specifically found in the two clusters indicated that anesthesia improved the response of CD4^+^ Th cells cytokines and the cytokines’ production (Fig. 3I).

**Fig. 4:**
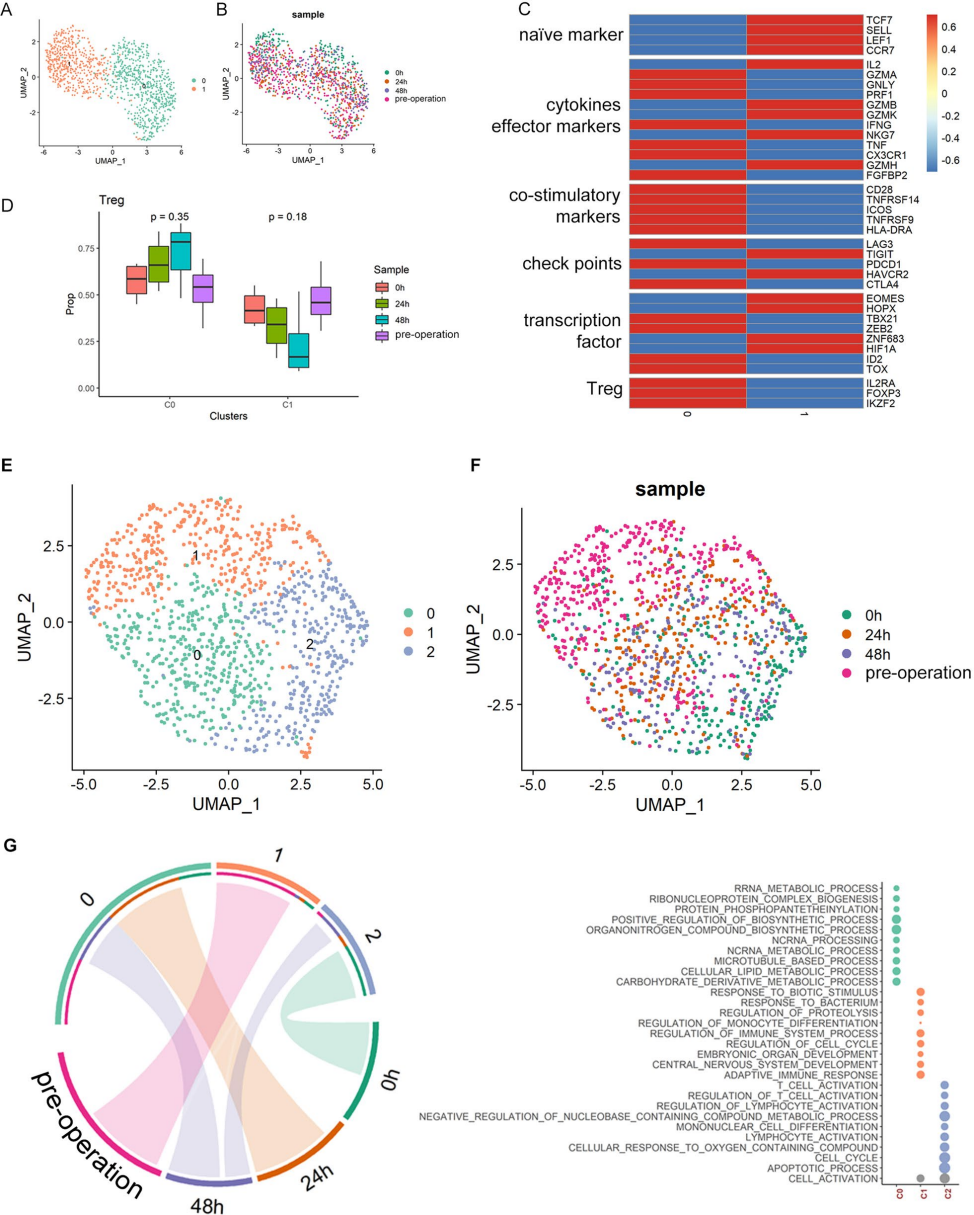
Clustering of Treg cells during the perioperative period and scSTAR reveals a stimulus pattern of anesthesia-related Treg cells dynamics. **A** UMAP plots of Treg cells which were classified as 2 clusters. **B** UMAP plots of Treg cells from 4 time points. **C** Heatmap of Z-score normalized log2(count + 1) expression of selected T cell function-associated genes in each cell cluster. **D** Changes of proportions of c0 and c1 in Treg cells during the perioperative period. **E** UMAP plot of 2 cell clusters processed with scSTAR. **F** UMAP plot of samples from 4 time points. **G** The association between scSTAR cell clustering results and time points using hypergeometric test. H. Biological process enriched from marker genes of C0–C2

The 1131 Treg cells were categorized into two clusters (Fig. 4A, B and Table S7). Based on the expression of canonical markers (Fig. 4C), C0 was annotated as effector Treg (eTreg) as they had high expression of FOXP3 but low naïve markers, e.g., SELL, LEF1, TCF7. On the contrary, C1 had high expression of naïve markers but low FOXP3, therefore, this cluster was annotated as naïve Treg (nTreg). By interrogating the Treg clusters in different time points, we concluded that anesthesia did not affect Treg cluster compositions (Fig. 4D).

Similar to Th cells, the Treg cell dynamic revealed by scSTAR also demonstrated a quick response pattern (Fig. 4E, F; Table S8). The response patterns can be categorized into two stages, i.e., 0 and 24–48 h (Fig. 4G). GO analysis results indicated that the immune regulation function of Treg cells were activated at 0 h (Fig. 4H). Taking the activation of Th cells at 0 h into consideration, the activity of Treg cells might be regarded as a counteract of immune activation. The functions of Treg continued to change during 24–48 h post operation, during which, energy related functions were more active (Fig. 4H).

### Monocytes exhibited a delayed yet pro-tumoral response  

**Fig. 5:**
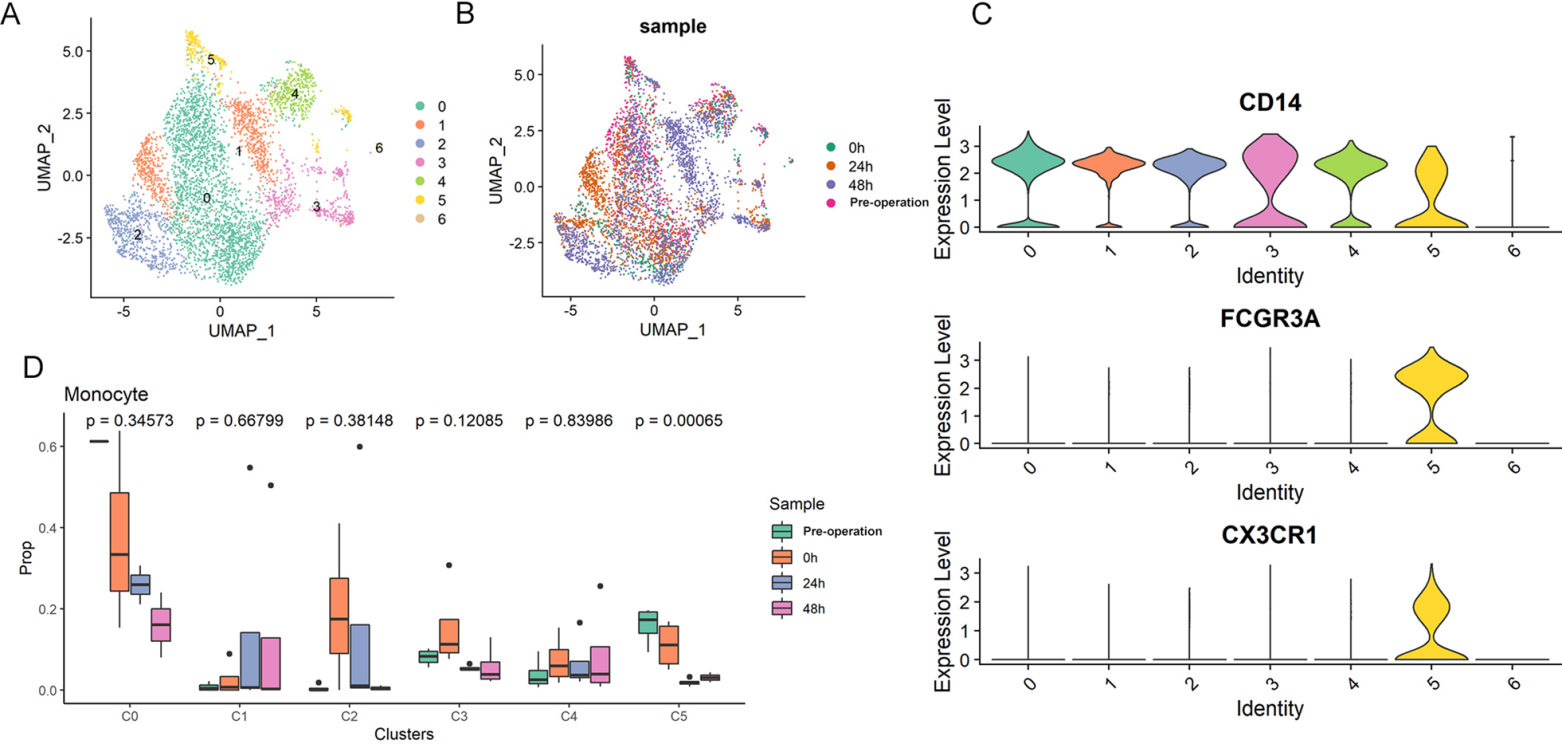
Clustering of monocytes during the perioperative period. **A** UMAP plots of monocytes which were classified as 7 clusters. **B** UMAP plots of monocytes from 4 time points. **C** CD14, FCGR3 A(CD16), and CX3 CR1 expression level in all clusters of monocytes. **D** Changes of proportions of C0, C1, C2, C3, C4, and C5 in Treg cells during the perioperative period

By analyzing the expression data, we can find that the monocyte can be divided into 7 clusters (Fig. 5A, B and Table S9). Accord the expression patterns of CD14, FCGR3 A, and CX3 CR1, we annotated C0–C4 as classical monocyte and C5 as non-classical monocyte (Fig. 5C). The time point compositions of different clusters were illustrated in Fig. 5D, which illustrates that only the proportions of non-classical monocyte (C5) significantly decreased during perioperative period. Previous studies illustrated that classical monocytes promote tumorigenesis and non-classical monocyte may reduce the tumor metastasis (Hanna et al. 2015; Landhuis 2016), and the effect of non-classical monocyte was achieved through the recruitment and activation of NK cells(Narasimhan et al. 1950). Therefore, the antitumoral capability may drop as the decrease of non-classical monocytes after anesthesia.

**Fig. 6:**
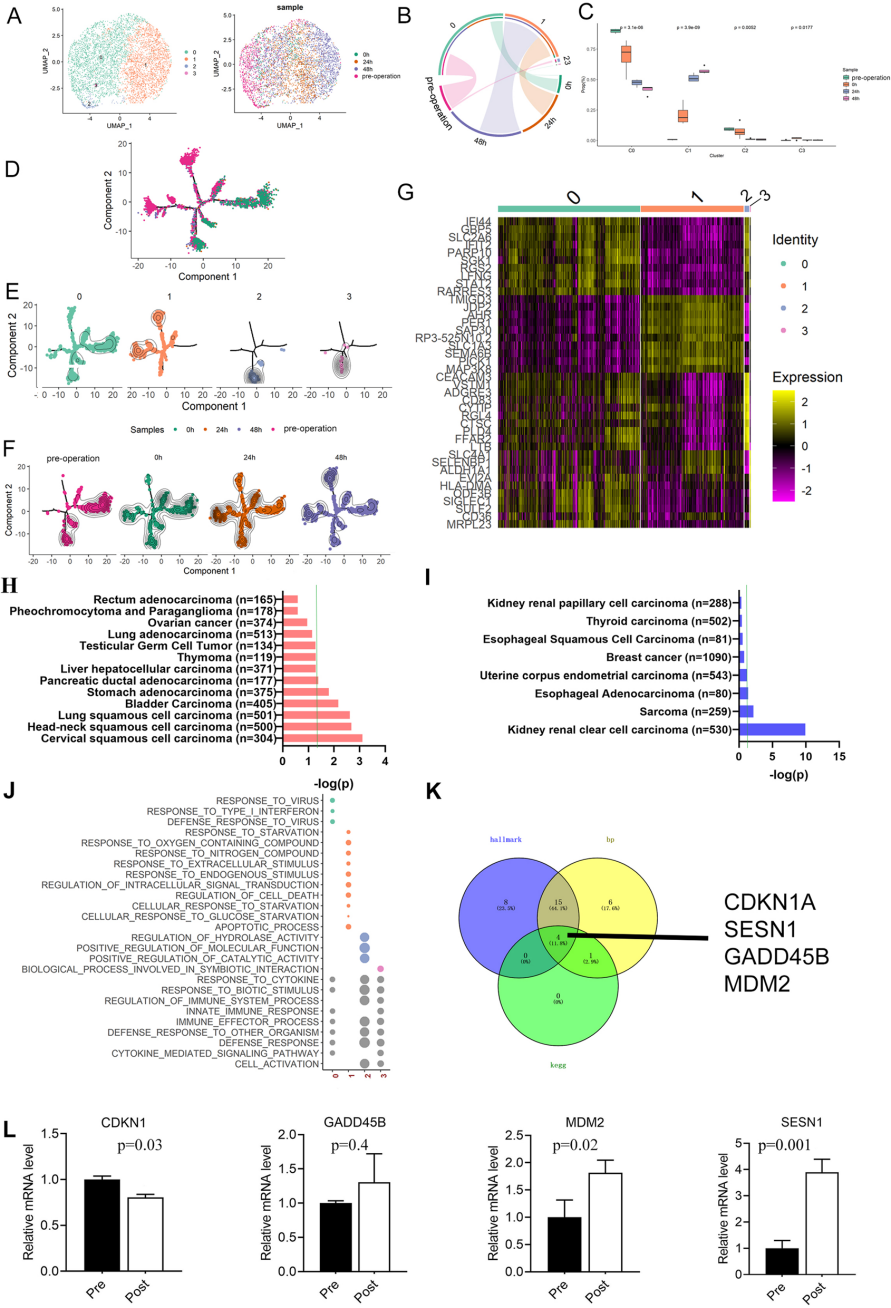
scSTAR reveals a stimulus pattern of anesthesia-related monocytes dynamics. **A** t-SNE plot of 4 cell clusters and samples from 4 time points processed with scSTAR. **B** The association between scSTAR cell clustering results and time points using hypergeometric test. **C** The relationship between clusters 0–3 and the different time groupings. **D**–**F** Pseudotime trajectory illustrates the transition and differentiation of monocyte clusters from pre-operation to post-operation 48 h with scSTAR processed data. **F** Heatmap of some differently expressed genes of monocyte clusters with scSTAR processed data. **G** The relationship between the expression of C0 characteristic genes and the survival time of common tumor patients, and negative sign represents tumor suppression. **H** The relationship between the expression of C1 characteristic genes and the survival time of common tumor patients, and negative sign represents tumor suppression. **I**. Biological process enriched from genes in Fig. 6 F. **J** Common genes on hallmark, biological process and KEGG pathway enriched from differently expressed genes of monocyte clusters with scSTAR. **K** Changes of CDKN1 A, SESN1, GADD45B, and MDM2 in tumor patients subjected to surgery between before and after surgery

By analyzing the scSTAR processed data, we found that monocyte could be divided into 4 clusters (C0–C3), and these 4 clusters showed obvious temporal characteristics (Fig. 6A; Table S10). As shown in Fig. 6B, C0 was dominated by preoperative samples at control and 0 h post operation, C1 was mainly derived from samples at 24 h and 48 h post operation, c2 was mainly derived from preoperative samples, and C3 was mainly derived from samples at 0 h post operation. However, the proportions of C2 and C3 cells were extremely low (Fig. 6B, C). Pseudotime trajectory analysis found (Fig. 6D–F) that cells from control and 0 h post operation samples have certain level of overlap, however, 24 and 48 h cells showed more distinct distribution patterns on the trajectory. It implied that monocytes have slow but profound response to anesthesia.

By analyzing the relationship between expression level of marker genes in C1 with the survival curve of cancer patients (Fig. S1) (Nagy et al. 2021), we found that high expression of these genes would shorten the survival time of most tumor patients and showed obvious cancer-promoting effects (Fig. 6G–I). The results of GO analysis confirmed the effect of monocyte on tumor cells. The results show that response to type I interferon was enriched in C0 pre-operation (Fig. 6J). Studies have shown that interferon I can enhance the activation of monocyte precursors (Santini et al. 2000), promote the conversion of monocyte to DC (Santodonato et al. 1950), and play an anti-tumor effect. The results of enrichment in C1 post operation showed that C1 marker genes were enriched in response to starvation, cellular response to starvation, and cellular response to glucose starvation. Under starvation, the number of monocytes decreased and the level of chronic inflammation decreased (Jordan et al. 2019). The increase in this anti-inflammatory effect in the tumor microenvironment promotes tumor growth (Rasheed and Rayner 2021). The C1 genes were enriched in regulation of cell death, in which entotic cell death promotes aneuploidy and polyploidy, thereby promoting tumor development (Galluzzi et al. 2018). At the same time, the enriched biological process also included the apoptosis process, which was actually a kind of regulated cell death. Studies have shown that tumors can promote the maturation and early apoptosis of monocyte-derived dendritic cells, thereby affecting the effect of immunotherapy. It can be seen that the apoptosis of monocyte promotes the development of tumor (Kiertscher et al. 2000; Santana-Magal et al. 2020).

Then we enriched the marker genes of C1 for biological functions. We found that 27 of these genes can be enriched to related hallmark gene sets, 36 genes enriched to related biological processes, and 5 genes enriched to related KEGG metabolic pathway. The intersection of these genes in the three types of bioinformatics analysis contained four genes: CDKN1 A, SESN1, GADD45B, and MDM2 (Fig. 6K) calculated with Venny 2.1 (Oliveros).

Then we have isolated monocytes from the blood of other three patients with lung cancer, and used RT-PCR technology to determine their expression levels. We found that the expression of MDM2, SESN1 increased obviously after surgery, which was consistent with the previous single-cell sequencing results. Another gene, GADD45B, showed an increasing trend but with no significant different (Fig. 6L). Among them, the MDM2 was known as a negative regulator of tumor suppressor p53 (Venkatesh et al. 2020).

## Discussion

In this study, by analyzing the scRNA-seq data of PBMC at 4 time points in the perioperative period, it was found that the lymphoid cell cluster represented by CD4^+^ T cells showed rapid response; and myeloid cell clusters represented by monocyte showed slow but profound response. Furthermore, it was speculated the alterations in monocyte might have pro-tumoral properties.

The effect of surgery/anesthesia on CD4^+^ and CD8^+^ T lymphocytes was temporary. However, another study showed that T lymphocyte apoptosis increased in patients using sevoflurane (Loop et al. 2005). This may be because when we evaluated the changes in the proportion of T cells at 4 time points. Meanwhile, another study had found that similar to ours, and general anesthesia and spinal anesthesia had little effect on the proportion of lymphocytes following surgery (Koksoy et al. 2013). There were relatively few studies on the functional status of T lymphocytes. This was an important finding of this study, and it was also a phenomenon not found in previous related studies. Based on this, we speculated that although CD4^+^ Th and CD8^+^ T cells were affected by general anesthesia surgery, which had limited impact on the long-term prognosis of patients.

Similar to CD4^+^ Th and CD8^+^ T cells, the proportion of Treg cells did not change significantly during the perioperative period, which was consistent with previous research findings (Oh et al. 2018). But another study found that the proportion of Tregs decreased significantly 7 days after surgery (Wang et al. 2016). Studies had shown that sevoflurane did have small effect on Treg, but the use of desflurane can significantly increase the proportion of Treg at 24 h post operation (Chutipongtanate et al. 2020). However, the changes in Treg cell function had not recovered at 48 h post operation in our study, while there were relatively few studies on changes in Treg function during the perioperative period.

Surgery/anesthesia had a longer lasting effect on monocytes, and led cancer-promoting gene expression. In this study, the functional changes of monocytes showed that the tumor suppressor properties of marker genes were decreasing, and the cancer-promoting properties were increasing. That study demonstrated that postoperative epidural analgesia retained the function of lymphocytes instead of monocytes, and the function of monocytes declines after surgery (Volk et al. 2004). The gene expression changes in monocytes caused by surgery/anesthesia might tend to shorten the survival time of tumor patients. This has not been reported before, and it was also the main finding of this study. More researches are needed on their rely on pathway or deeper mechanism.

Previous studies have shown that in monocytes, MDM2 can bind to p53 to inhibit the anti-tumor activity, while MDM2 can bind to p53 to inhibit the killing (Namgaladze and Brüne 2021) effect of NK cells on tumor cells (Veneziani et al. 2021). At present, a number of anti-tumor drugs targeting MDM2 have been developed (Konopleva et al. 2020), including APG-115 (Fang et al. 2019), KRT-232 (Zhang et al. 2020), and ALRN-6924 (Pairawan et al. 2021). We believe inhibition of MDM2 activity can improve the long-term prognosis of tumor patients undergoing general anesthesia surgery. This might be the target of our future treatment to improve the immune function of cancer patients.

## Limitation

Fasting before surgery and hunger after surgery are part of anesthesia/surgery. Generally speaking, most surgical patients are still hungry 24 h after surgery. We tried to observe the overall effect of anesthesia/surgery. This study collected data at 4 time points in the perioperative period, and the number of patients included in the single-cell sequencing was relatively small, with only 4 patients. Here, this research might provide valuable information for analyzing larger sample sets in a more economical, effective, and direct way like a preliminary experiment.

## Supplementary Information

Additional file 1.

Additional file 2. Table S3

Additional file 3. Table S4

Additional file 4. Table S5

Additional file 5. Table S6

Additional file 6. Table S7

Additional file 7. Table S8

Additional file 8. Table S9

Additional file 9. Table S10

## Data Availability

Data is provided within the manuscript or supplementary information files.

## References

[CR1] Buggy DJ, Borgeat A, Cata J, Doherty DG, Doornebal CW, Forget P, et al. Consensus statement from the BJA workshop on cancer and anaesthesia. Br J Anaesth. 2015;114:2–3. 25104229 10.1093/bja/aeu262

[CR2] Chutipongtanate A, Prukviwat S, Pongsakul N, Srisala S, Kamanee N, Arpornsujaritkun N, et al. Effects of Desflurane and Sevoflurane anesthesia on regulatory T cells in patients undergoing living donor kidney transplantation: a randomized intervention trial. BMC Anesthesiol. 2020;20:215. 32854613 10.1186/s12871-020-01130-7 PMC7450591

[CR3] Fang DD, Tang Q, Kong Y, Wang Q, Gu J, Fang X, et al. MDM2 inhibitor APG-115 synergizes with PD-1 blockade through enhancing antitumor immunity in the tumor microenvironment. J Immunother Cancer. 2019;7:327. 31779710 10.1186/s40425-019-0750-6 PMC6883539

[CR4] Galluzzi L, Vitale I, Aaronson SA, Abrams JM, Adam D, Agostinis P, et al. Molecular mechanisms of cell death: recommendations of the nomenclature committee on cell death 2018. Cell Death Differ. 2018;25:486–541. 29362479 10.1038/s41418-017-0012-4 PMC5864239

[CR5] Guo X, Zhang Y, Zheng L, Zheng C, Song J, Zhang Q, et al. Global characterization of T cells in non-small-cell lung cancer by single-cell sequencing. Nat Med. 2018;24:978–85. 29942094 10.1038/s41591-018-0045-3

[CR6] Hanna RN, Cekic C, Sag D, Tacke R, Thomas GD, Nowyhed H, et al. Patrolling monocytes control tumor metastasis to the lung. Science (New York, NY). 2015;350:985–90. 10.1126/science.aac9407 PMC4869713 26494174

[CR7] Jie H, Ke C, Jiaqiang Z, Duojiao W, Wei C, Jiawei Z et al. scSTAR reveals hidden heterogeneity with a real-virtual cell pair structure across single cell samples. Research Square. 2022.

[CR8] Jordan S, Tung N, Casanova-Acebes M, Chang C, Cantoni C, Zhang D, et al. Dietary intake regulates the circulating inflammatory monocyte pool. Cell. 2019;178:1102-14.e17. 31442403 10.1016/j.cell.2019.07.050 PMC7357241

[CR9] Juneja R. Opioids and cancer recurrence. Curr Opin Support Palliat Care. 2014;8:91–101. 24759319 10.1097/SPC.0000000000000056

[CR10] Kiertscher SM, Luo J, Dubinett SM, Roth MD. Tumors promote altered maturation and early apoptosis of monocyte-derived dendritic cells. J Immunol. 2000;164:1269–76. 10640740 10.4049/jimmunol.164.3.1269

[CR11] Koksoy S, Sahin Z, Karsli B. Comparison of the effects of desflurane and bupivacaine on Th1 and Th2 responses. Clin Lab. 2013;59:1215–20. 24409654 10.7754/clin.lab.2013.120413

[CR12] Konopleva M, Martinelli G, Daver N, Papayannidis C, Wei A, Higgins B, et al. MDM2 inhibition: an important step forward in cancer therapy. Leukemia. 2020;34:2858–74. 32651541 10.1038/s41375-020-0949-z

[CR13] Landhuis E. Innate immune cells may prevent metastasis. Cancer Discov. 2016;6:6–7. 26573661 10.1158/2159-8290.CD-NB2015-155

[CR14] Loop T, Dovi-Akue D, Frick M, Roesslein M, Egger L, Humar M, et al. Volatile anesthetics induce caspase-dependent, mitochondria-mediated apoptosis in human T lymphocytes in vitro. Anesthesiology. 2005;102:1147–57. 15915027 10.1097/00000542-200506000-00014

[CR15] Müller SD, Ziegler JSH, Piegeler T. Local anesthetics and recurrence after cancer surgery-what's new? A narrative review. J Clin Med. 2021;10. 10.3390/jcm10040719 PMC7918400 33670434

[CR16] Nagy Á, Munkácsy G, Győrffy B. Pancancer survival analysis of cancer hallmark genes. Sci Rep. 2021;11:6047. 33723286 10.1038/s41598-021-84787-5 PMC7961001

[CR17] Namgaladze D, Brüne B. Pharmacological Activation of p53 during human monocyte to macrophage differentiation attenuates their pro-inflammatory activation by TLR4, TLR7 and TLR8 Agonists. Cancers. 2021;13. 10.3390/cancers13050958 PMC7956237 33668835

[CR18] Narasimhan PB, Eggert T, Zhu YP, Marcovecchio P, Meyer MA, Wu R, et al. Patrolling monocytes control NK cell expression of activating and stimulatory receptors to curtail lung metastases. J Immunol (Baltimore, Md : 1950). 2020;204:192–8. 10.4049/jimmunol.1900998 PMC7890694 31767784

[CR19] Oh CS, Lee J, Yoon TG, Seo EH, Park HJ, Piao L, et al. Effect of equipotent doses of propofol versus sevoflurane anesthesia on regulatory T cells after breast cancer surgery. Anesthesiology. 2018;129:921–31. 30074934 10.1097/ALN.0000000000002382

[CR20] Oliveros JCV-. An interactive tool for comparing lists with Venn's diagrams.

[CR21] Pairawan S, Zhao M, Yuca E, Annis A, Evans K, Sutton D, et al. First in class dual MDM2/MDMX inhibitor ALRN-6924 enhances antitumor efficacy of chemotherapy in TP53 wild-type hormone receptor-positive breast cancer models. Breast Cancer Res BCR. 2021;23:29. 33663585 10.1186/s13058-021-01406-x PMC7934277

[CR22] Perry NJS, Buggy D, Ma D. Can anesthesia influence cancer outcomes after surgery? JAMA Surg. 2019;154:279–80. 30649136 10.1001/jamasurg.2018.4619

[CR23] Rasheed A, Rayner KJ. Macrophage responses to environmental stimuli during homeostasis and disease. Endocr Rev. 2021;42:407–35. 33523133 10.1210/endrev/bnab004 PMC8284619

[CR24] Santana-Magal N, Farhat-Younis L, Gutwillig A, Gleiberman A, Rasoulouniriana D, Tal L, et al. Melanoma-secreted lysosomes trigger monocyte-derived dendritic cell apoptosis and limit cancer immunotherapy. Can Res. 2020;80:1942–56. 10.1158/0008-5472.CAN-19-2944 32127354

[CR25] Santini SM, Lapenta C, Logozzi M, Parlato S, Spada M, Di Pucchio T, et al. Type I interferon as a powerful adjuvant for monocyte-derived dendritic cell development and activity in vitro and in Hu-PBL-SCID mice. J Exp Med. 2000;191:1777–88. 10811870 10.1084/jem.191.10.1777 PMC2193160

[CR26] Santodonato L, D'Agostino G, Nisini R, Mariotti S, Monque DM, Spada M, et al. Monocyte-derived dendritic cells generated after a short-term culture with IFN-alpha and granulocyte-macrophage colony-stimulating factor stimulate a potent Epstein-Barr virus-specific CD8+ T cell response. Journal of immunology (Baltimore, Md: 1950). 2003;170:5195–202. 10.4049/jimmunol.170.10.5195 12734367

[CR27] Veneziani I, Infante P, Ferretti E, Melaiu O, Battistelli C, Lucarini V, et al. Nutlin-3a enhances natural killer cell-mediated killing of neuroblastoma by restoring p53-dependent expression of ligands for NKG2D and DNAM-1 receptors. Cancer Immunol Res. 2021;9:170–83. 33303573 10.1158/2326-6066.CIR-20-0313

[CR28] Venkatesh D, O’Brien NA, Zandkarimi F, Tong DR, Stokes ME, Dunn DE, et al. MDM2 and MDMX promote ferroptosis by PPARα-mediated lipid remodeling. Genes Dev. 2020;34:526–43. 32079652 10.1101/gad.334219.119 PMC7111265

[CR29] Volk T, Schenk M, Voigt K, Tohtz S, Putzier M, Kox WJ. Postoperative epidural anesthesia preserves lymphocyte, but not monocyte, immune function after major spine surgery. Anesthesia and analgesia. 2004;98:1086–92, table of contents. 10.1213/01.ANE.0000104586.12700.3A 15041604

[CR30] Wang XT, Lv M, Guo HY. Effects of epidural block combined with general anesthesia on antitumor characteristics of T helper cells in hepatocellular carcinoma patients. J Biol Regul Homeost Agents. 2016;30:67–77. 27049077

[CR31] Wang YY, Chang EQ, Zhu RL, Liu XZ, Wang GZ, Li NT, et al. An atlas of dynamic peripheral blood mononuclear cell landscapes in human perioperative anaesthesia/surgery. Clin Transl Med. 2022;12: e663. 35061932 10.1002/ctm2.663 PMC8782495

[CR32] Zhang X, Zhang R, Chen H, Wang L, Ren C, Pataer A, et al. KRT-232 and navitoclax enhance trametinib’s anti-Cancer activity in non-small cell lung cancer patient-derived xenografts with KRAS mutations. Am J Cancer Res. 2020;10:4464–75. 33415011 PMC7783771

[CR33] Zheng Y, Chen Z, Han Y, Han L, Zou X, Zhou B, et al. Immune suppressive landscape in the human esophageal squamous cell carcinoma microenvironment. Nat Commun. 2020;11:6268. 33293583 10.1038/s41467-020-20019-0 PMC7722722

