# Supplementary material for: Surgery/anesthesia may cause monocytes to promote tumor development
Source: Mol Med. 2025 May 7;31:178. doi: 10.1186/s10020-025-01213-6 (PMC12060369; doi:10.1186/s10020-025-01213-6)
Supplement: Supplementary file 1 — Additional file 1. [file 10020_2025_1213_MOESM1_ESM.pdf]

Table s1. Demographic characteristics of patients in the study

Items		1	2	3	4
Age(y)		17	28	62	63
Sex (M/F)		F	F	M	F
Height (cm)		161	163	171	159
Weight(kg)		52	55	65	62
Diagnosis		Ovarian teratoma	Ovarian teratoma	Meningioma	Knee osteoarthritis
Operation		Laparoscopic tumor resection	Laparoscopic tumor resection	Intracranial tumor resection	Knee replacement
Operation time		2 h	2 h	4h	2h
Incision type		Minor	Minor	Major	Major
Anesthetics		Propofol	Sevoflurane	Propofol + Sevoflurane	Propofol + Sevoflurane
WBC ($x10^9$/L)	Pro-	8.04	5.46	6.6(65.3%)	4.2
	Post-			9.6	
	24			11.4	11.79
	48			8.1	
Neutrophils ($x10^9$/L)	Pro-	5.62	2.63	4.33(8.4%)	2.1(49.7%)
	Post-			8.96(93.4%)	
	24			10.15(89.3%)	10.56(89.5%)
	48			6.05(74.7%)	
Monocytes ($x10^9$/L)	Pro-	0.6	0.45	8.4%	8.2%
	Post-			0.6%	
	24			3.2%	0.59(5%)
	48			4.5%	
Lymphocyte ($x10^9$/L)	Pro-	1.66	2.26	1.65(24.9%)	1.62(38.4%)
	Post-			0.57(5.9%)	
	24			0.82(7.2%)	0.62%（5.3）
	48			1.65(20.3%)	
Platelet ($x10^9$/L)	Pro-	294	440	178	205
	Post-			191	
	24			126	208
	48			156	
Complications		No	No	No	No
Prognosis		Good	Good	Good	Good

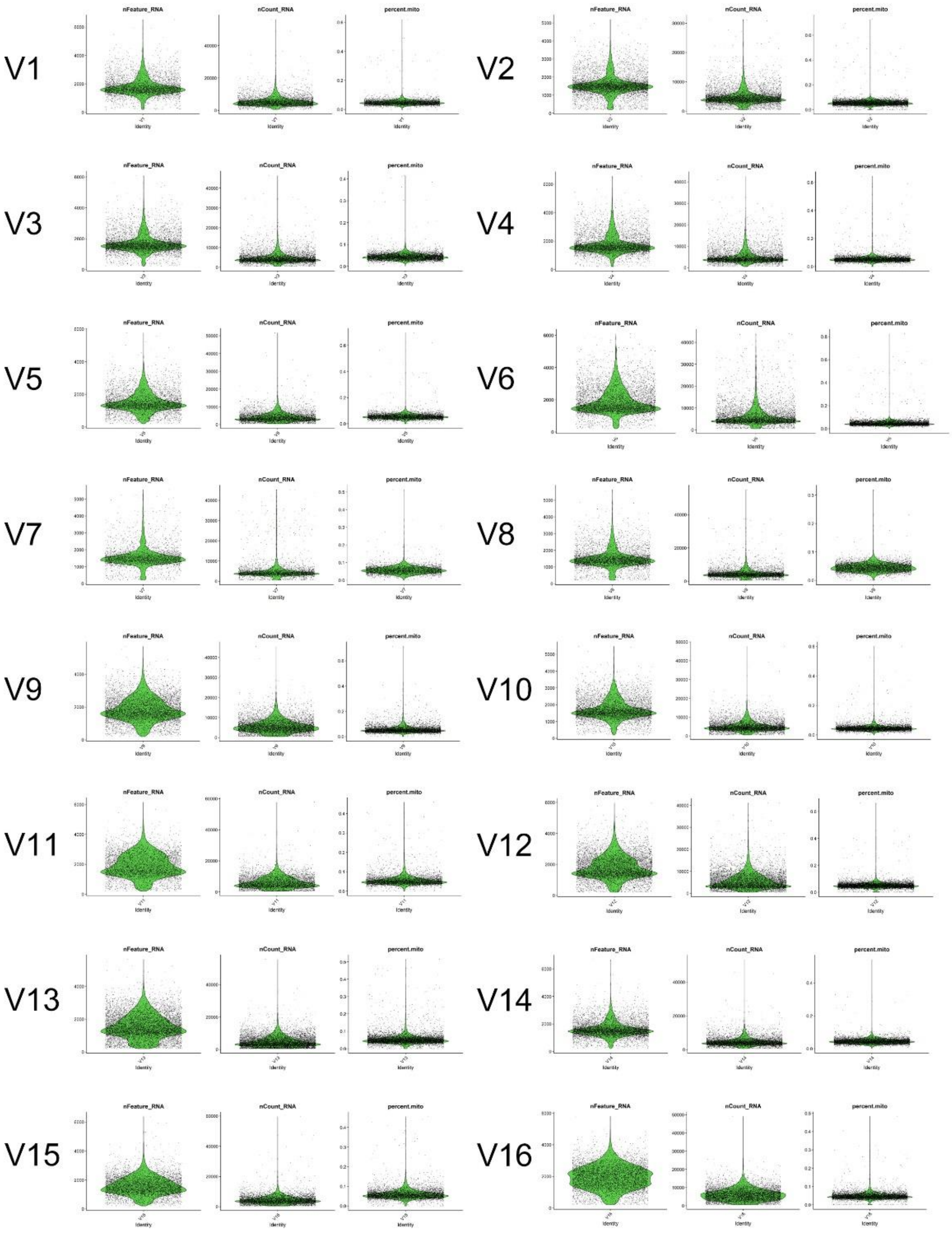

Figure QC. For all 16 sample, we used Seurat R package v4.0.3 to screen low quality cells. Specifically, the cells with 200<nFeature_RNA<4000, nCount_RNA<20000 and percent.mito<0.1 were preserved. The violin plots for quality control were shown.

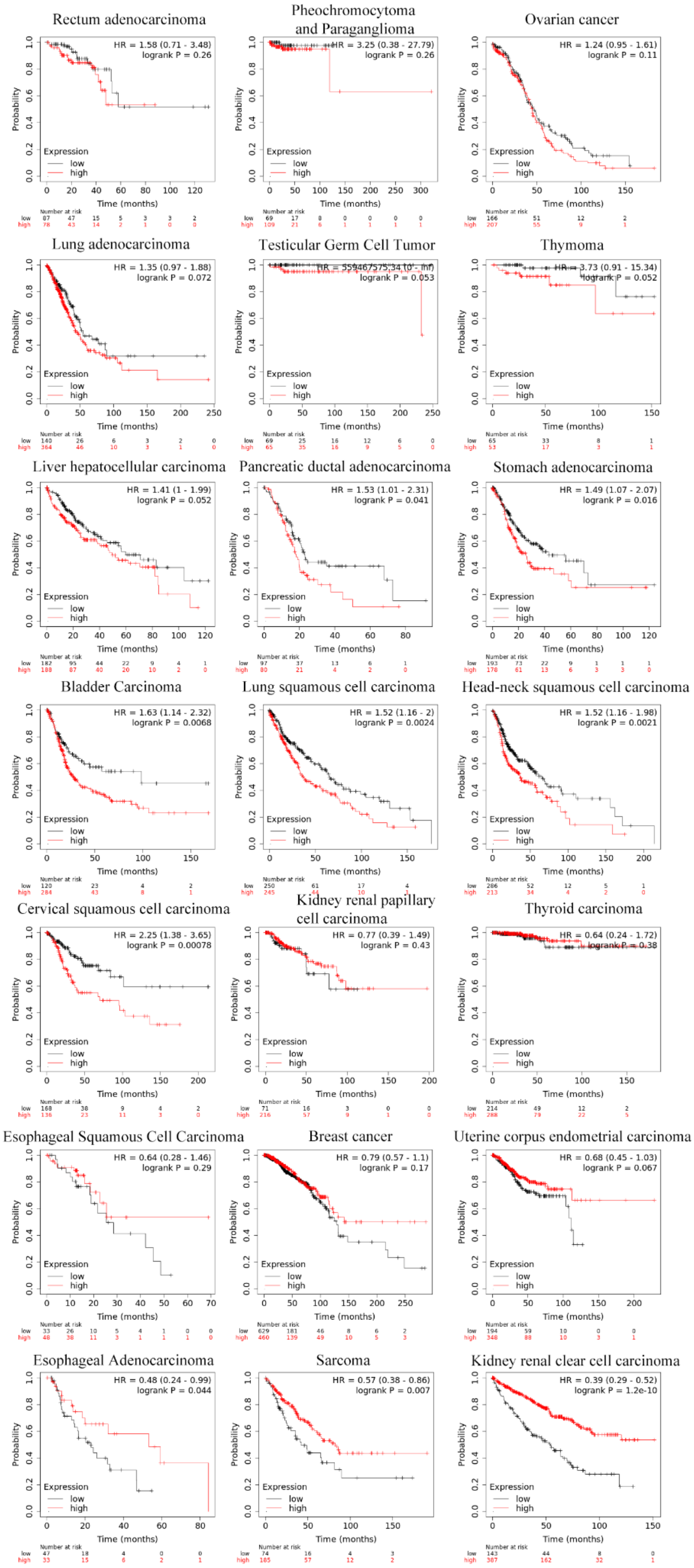

Rectum adenocarcinoma
HR = 1.58 (0.71 - 3.48)
logrank P = 0.26
Pheochromocytoma and Paraganglioma
HR = 3.25 (0.38 - 27.79)
logrank P = 0.26
Ovarian cancer
HR = 1.24 (0.95 - 1.61)
logrank P = 0.11
Lung adenocarcinoma
HR = 1.35 (0.97 - 1.88)
logrank P = 0.072
Testicular Germ Cell Tumor
logrank P = 0.053
Thymoma
HR = 3.73 (0.91 - 15.34)
logrank P = 0.052
Liver hepatocellular carcinoma
HR = 1.41 (1 - 1.99)
logrank P = 0.052
Pancreatic ductal adenocarcinoma
HR = 1.53 (1.01 - 2.31)
logrank P = 0.041
Stomach adenocarcinoma
HR = 1.49 (1.07 - 2.07)
logrank P = 0.016
Bladder Carcinoma
HR = 1.63 (1.14 - 2.32)
logrank P = 0.0068
Lung squamous cell carcinoma
HR = 1.52 (1.16 - 2)
logrank P = 0.0024
Head-neck squamous cell carcinoma
HR = 1.52 (1.16 - 1.98)
logrank P = 0.0021
Cervical squamous cell carcinoma
HR = 2.25 (1.38 - 3.65)
logrank P = 0.00078
Kidney renal papillary cell carcinoma
HR = 0.77 (0.39 - 1.49)
logrank P = 0.43
Thyroid carcinoma
HR = 0.64 (0.24 - 1.72)
logrank P = 0.38
Esophageal Squamous Cell Carcinoma
HR = 0.64 (0.28 - 1.46)
logrank P = 0.29
Breast cancer
HR = 0.79 (0.57 - 1.1)
logrank P = 0.17
Uterine corpus endometrial carcinoma
HR = 0.68 (0.45 - 1.03)
logrank P = 0.067
Esophageal Adenocarcinoma
HR = 0.48 (0.24 - 0.99)
logrank P = 0.044
Sarcoma
HR = 0.57 (0.38 - 0.86)
logrank P = 0.007
Kidney renal clear cell carcinoma
HR = 0.39 (0.29 - 0.52)
logrank P = 1.2e-10
Probability
Time (months)
Expression
low
high
Number at risk

Figure s1. Using the gene markers of cluster 1 as a gene set, the Kaplan-Meier plot shows the high expression of cluster 1 gene set has a worse survival in most cancers in TCGA.

Table s2 Primers' list

Gene symbol	Forward primer (5'-3')	Reverse primer (5'-3')
CDKN1A	CGATGGAACTTCGACTTTGTCA	GCACAAGGGTACAAGACAGTG
SESN1	TCACACACTATCATTCTCTTGCC	ACATTCCTGTAACTGCCTCATCT
MDM2	GGCAGGGGAGAGTGATACAGA	GAAGCCAATTCTCACGAAGGG
GADD45B	TACGAGTCGGCCAAGTTGATG	GGATGAGCGTGAAGTGGATTT
